# VISION IMPAIRMENT AND SOCIAL ISOLATION IN OLDER AFRICAN AMERICANS: THE IMPACT ON COGNITIVE DECLINE

**DOI:** 10.1093/geroni/igac059.2407

**Published:** 2022-12-20

**Authors:** Emily Hayes, Avalon White, Corinna Trujillo Tanner, Jeremy Yorgason

**Affiliations:** Brigham Young University--Provo, Provo, Utah, United States; Brigham Young University, Provo, Utah, United States; Brigham Young University, Provo, Utah, United States; Brigham Young University, Provo, Utah, United States

## Abstract

Evidence suggests a consistent correlation between vision impairments, social isolation, and cognitive decline. The National Eye Institute reports that African Americans have an increased risk of developing certain vision impairments such as cataracts, glaucoma, and diabetic retinopathy. At the same time, older African Americans often receive care from family members and this family care may act as a buffer against social isolation and resulting cognitive decline. Using data from 737 African Americans that participated in waves 5, 6, and 7 of the National Health and Aging Trends Study (NHATS), we explored associations between vision impairment, social isolation, and cognitive functioning. Results showed that vision impairment at round 5 was related to increased social isolation, and higher social isolation at round 5 was related to decreased delayed word recall scores at the same wave. No significant longitudinal associations were found between these constructs. Findings suggest that concurrent associations exist between sensory impairments, social isolation, and cognitive functioning, but that these relationships are not robust across time. Despite support provided by unpaid family caregivers, African American older adults with vision impairment are at an increased risk for concurrent social and cognitive challenges. It may be that family support of those with sensory impairments helps so that these impairments aren’t related to social isolation or cognitive functioning across time. Researchers and clinicians could benefit older African Americans with sensory impairments by providing and encouraging support during early stages of vision loss.

